# Peroneus Longus Tendon Graft in Extensor Mechanism Reconstruction After Total Knee Arthroplasty: A Viable Alternative

**DOI:** 10.7759/cureus.68801

**Published:** 2024-09-06

**Authors:** Luis Henrique Longo, Marcos Paulo T Vanzin, Luis Antonio R Bauer, Carolline Popovicz Nunes, Lucas Rezende, Letícia H Miyamoto, Seiji G Nakahashi

**Affiliations:** 1 Orthopaedics and Traumatology, Universidade Federal do Paraná, Hospital do Trabalhador, Curitiba, BRA

**Keywords:** extensor mechanism knee injuries, extensor mechanism repair, patellar tendon rupture, peroneus longus tendon graft, total knee arthroplasty

## Abstract

The rupture of the extensor mechanism following total knee arthroplasty (TKA) is a rare and severe complication that poses a significant challenge in knee surgery. The rupture can occur in the patellar or quadriceps tendons or due to patellar fractures, leading to substantial functional deficits. Risk factors include multiple prior surgeries, systemic conditions, as well as iatrogenic injuries. Reconstruction techniques range from autografts, allografts, and synthetic materials to local flaps, but none have shown exceptional results. This case report describes extensor mechanism reconstruction using a peroneus longus tendon graft in a patient. A 74-year-old active female patient was referred from a rural area to a knee surgery referral hospital with a history of progressive osteoarthritis in the left knee and underwent TKA. At her first post-surgical follow-up, she was diagnosed with an extensor mechanism rupture and referred to our institution, being admitted six weeks postoperatively. On initial assessment, she presented difficulty in fully extending the knee and patellar elevation. Extensor mechanism reconstruction was indicated, using an autograft from the peroneus longus tendon due to limited access to tissue banks and synthetic materials. The graft was fixed using anchors and an interference screw, resulting in stable patellar tracking. Postoperatively, the patient followed a rehabilitation protocol with the use of a brace and gradual weight-bearing and mobility. One year after surgery, she demonstrated an unassisted gait without a limp, with restored extensor mechanism competence and a range of motion of 0-120 degrees. Several techniques have been described for patellar tendon reconstruction following TKA, but the results remain unsatisfactory. Most techniques involve the use of allografts, which, although moderately effective, can lead to residual deficits and the need for ambulatory aids. Synthetic materials have gained popularity but face challenges such as cost and tissue reactions. The use of autografts, like the peroneus longus tendon, has shown promise due to its robustness and adaptability. This report highlights a successful case of extensor mechanism reconstruction using the peroneus longus tendon. The peroneus longus tendon graft offers a viable alternative for extensor mechanism reconstruction after TKA: minimizes costs, reduces risks of tissue rejection and cross-infection, and facilitates early rehabilitation.

## Introduction

The rupture of the extensor mechanism following total knee arthroplasty (TKA) is a rare but potentially devastating complication, representing a significant challenge in knee surgery. The disruption can occur at the patellar or quadriceps tendon or in cases of patellar fractures as well [1]. Inadequate repair of the extensor mechanism results in significant functional deficits, affecting mobility and directly impacting the quality of life of these patients [2,3]. The incidence of these injuries following TKA is possibly underreported, but it is estimated that 0.1-1.1% affect the quadriceps tendon [4] and 0.17-1.4% involve the patellar tendon [5].

Among the risk factors for extensor mechanism rupture following TKA, multiple prior surgeries on this joint (average of three or four) are recognized as the primary predisposing factor in several studies [6-8]. However, systemic conditions such as chronic kidney disease, diabetes mellitus, obesity, and rheumatoid arthritis also contribute to the occurrence of the injury [7]. Another cause of extensor mechanism rupture in TKA is iatrogenic injuries, especially when attempting to gain exposure in a stiff knee [9].

Techniques for extensor mechanism reconstruction include the use of autografts [3], allografts [10], synthetic materials [11], and local flaps. Thus far, no method has shown satisfactory results, with improvements attributed to the evolution of surgical techniques themselves. The scarcity in the literature on this topic highlights the difficulty and, nonetheless, the need to pursue more satisfactory outcomes for extensor mechanism reconstruction [1].

This case report aims to present a unique experience of extensor mechanism reconstruction following TKA, using an autograft from the peroneus longus tendon in a patient referred to our institution. We present here the intraoperative findings and the outcomes of the method used.

## Case presentation

A 74-year-old, active, female patient was referred from a rural area to a knee surgery referral hospital. She had a past medical history of progressive knee osteoarthritis in the left knee, with deteriorating functional capacity and loss of independence in activities. She reported only systemic arterial hypertension as an associated comorbidity.

She began orthopedic follow-up in her hometown, undergoing conservative measures for knee osteoarthritis: weight loss, restriction of impact activities, use of chondroprotective agents, physiotherapy, and muscle strengthening. However, due to disease progression, the patient underwent TKA. According to reports, at her first follow-up appointment (two weeks post-surgery), she was referred to our institution due to extensor mechanism rupture following TKA.

The patient was admitted to our institution at the sixth week postoperatively (a delay of four weeks since referral). On initial physical examination, she ambulated with the assistance of a walker, with no weight-bearing on the left lower limb. The knee joint demonstrated an inability to fully extend, maintaining flexion of approximately 15-20 degrees, with a palpable gap at the patellar tendon site. There were no signs of inflammation at the surgical wound, and the scar appeared to be healing well. Preoperative laboratory tests did not show an increase in inflammatory markers.

**Figure 1:**
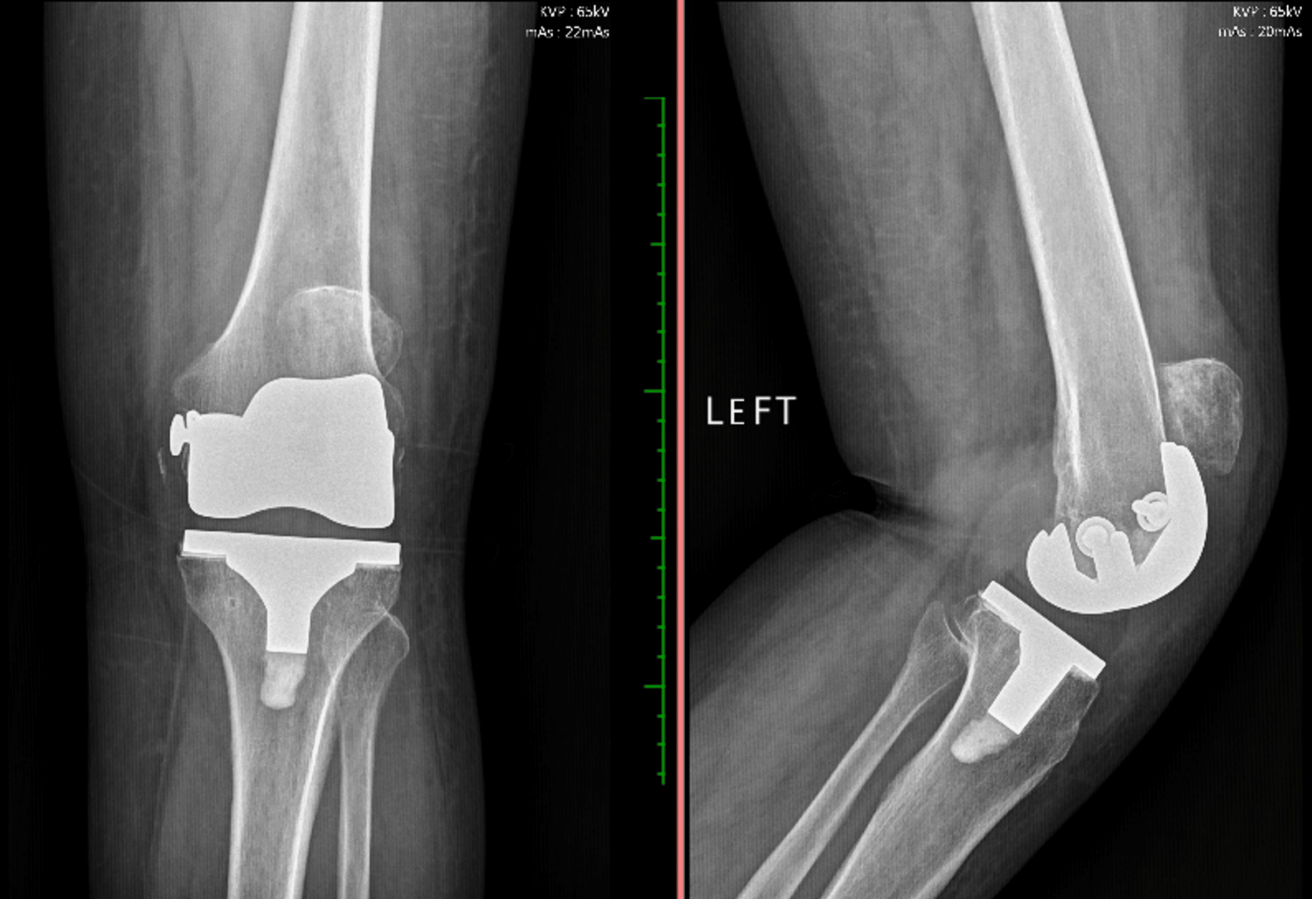
Knee radiograph in AP and lateral views, showing patellar elevation. AP: anteroposterior

The radiograph confirmed the findings of the physical examination, showing patellar elevation without signs of implant loosening, along with fixation of the medial femoral epicondyle, possibly secondary to an intraoperative fracture during TKA. These findings are illustrated in Figure 1.

After the initial assessment, surgical treatment was indicated for extensor mechanism reconstruction. Considering the difficulty in accessing tissue banks and the use of synthetic materials at our institution, we opted for an autograft for the procedure. Furthermore, in view of the TKA performed at another institution and the lack of information regarding the previous intraoperative details, we opted to use the peroneus longus graft to preserve the medial ligamentous balance of the knee and the hamstring tendons.

**Figure 2:**
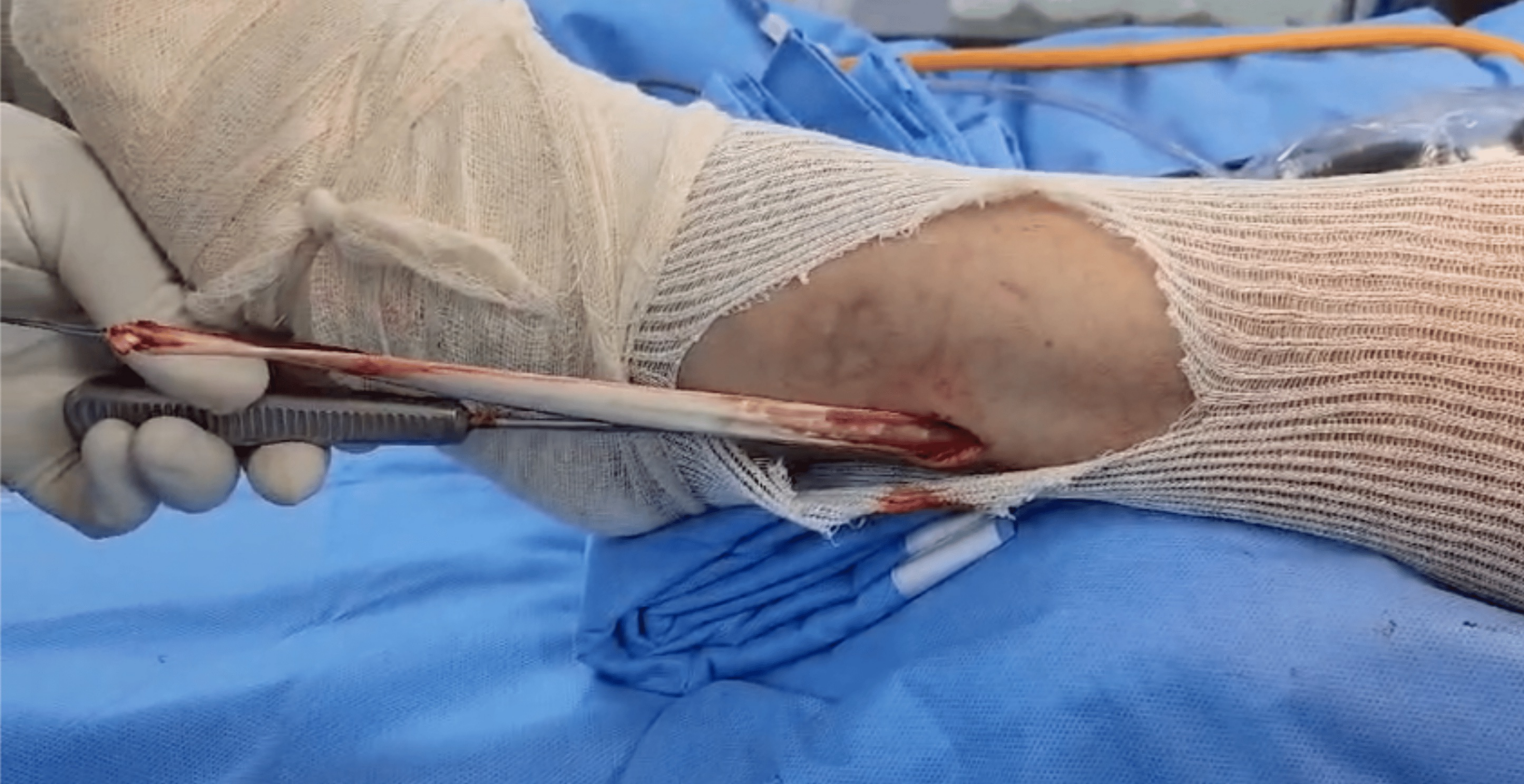
Harvesting of the long fibular graft for extensor mechanism reconstruction.

The flexor tendons are secondary restrictors of valgus stress, and their use may loosen the medial ligamentous balance. Additionally, the peroneus longus tendon has a favorable diameter and length for this purpose. Initially, the peroneus longus graft was harvested (Figure 2) followed by preparation for extensor mechanism reconstruction.

**Figure 3:**
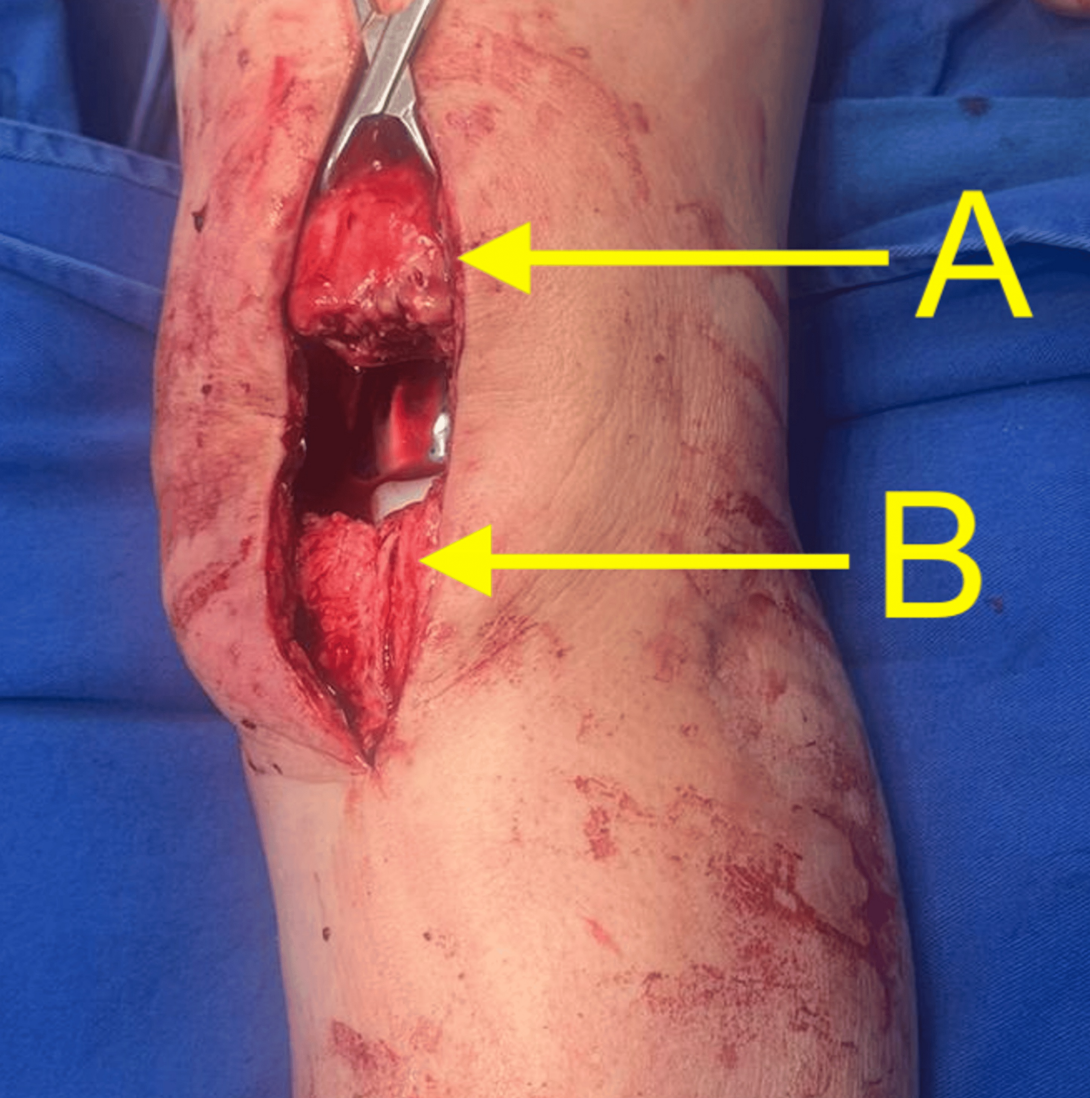
Visualization of left knee extensor mechanism rupture: (A) elevated patella and (B) ruptured patellar tendon.

Subsequently, access was achieved through a previous midline anterior incision, revealing the patellar tendon injury and patellar elevation. No signs of fibrosis, thickening, or other findings suggestive of previous patellar tendinopathy were observed, although the possibility of iatrogenic injury during primary knee arthroplasty cannot be ruled out (Figure 3).

After debridement of the extensor mechanism and collection of intraoperative cultures, patellar pegs and a tibial tunnel were created below the prosthetic component. The peroneus longus graft was fixed and tensioned using two proximal infrapatellar metal anchors adjacent to the rupture, along with an interference screw in the distal tunnel, achieving satisfactory coaptation of the extensor mechanism.

**Figure 4:**
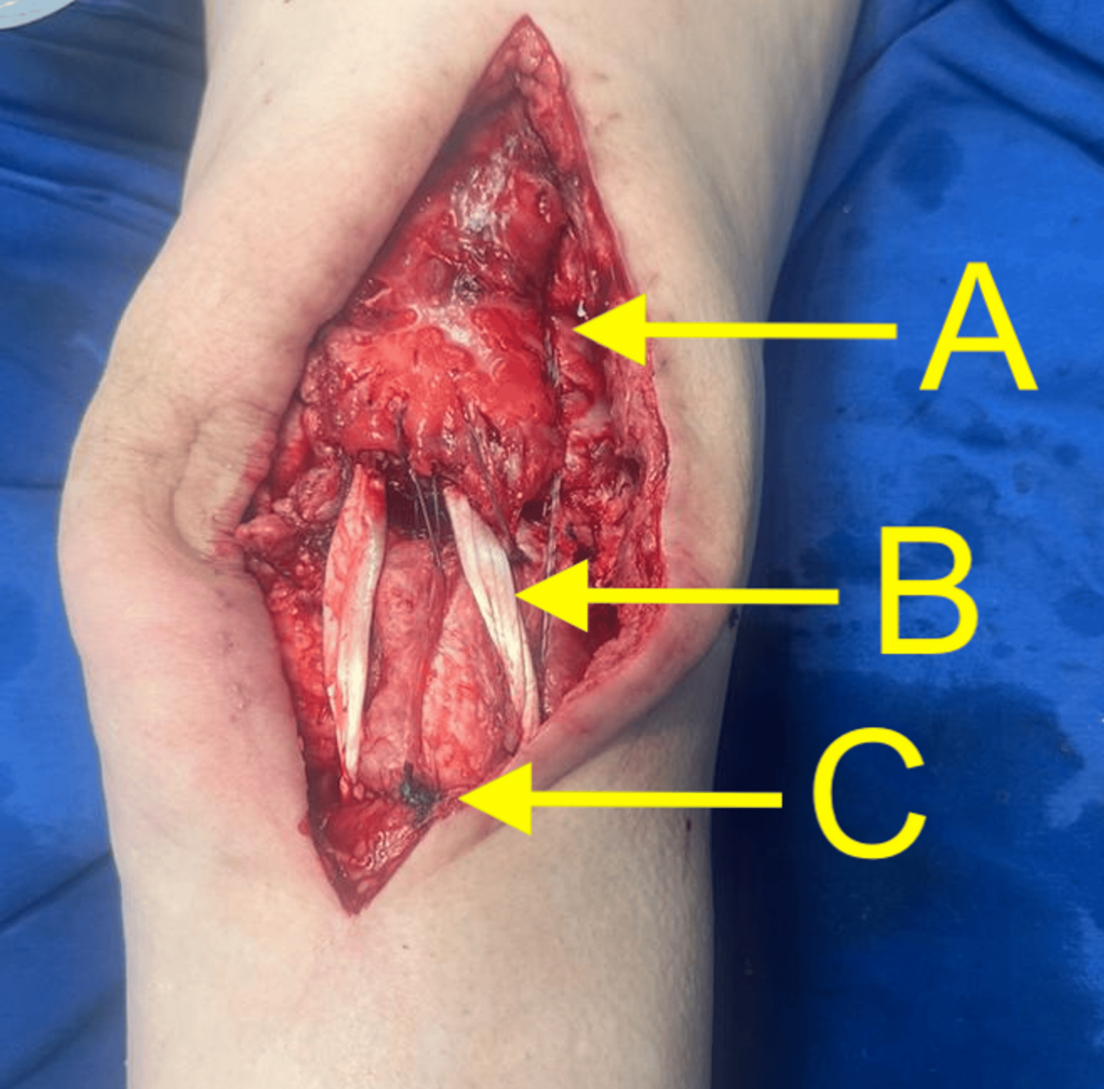
Extensor mechanism reconstructed: (A) high-strength sutures interlacing the retinaculum and quadriceps tendon, (B) autologous graft from the long fibular tendon fixed with two proximal infrapatellar metal anchors and a distal interference screw, and (C) metal anchor supporting high-strength sutures.

Subsequently, a third metal anchor was adapted in the region of the anterior tibial tuberosity, interlacing the retinacular structures and the quadriceps tendon with high-strength sutures, resulting in stable patellar tracking under direct visualization. Figure 4 depicts the appearance of the extensor mechanism after the completion of the reconstruction.

**Figure 5:**
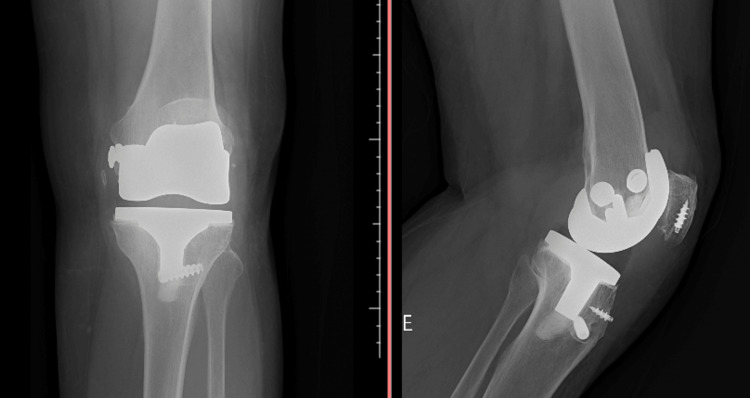
﻿﻿Knee radiograph in AP and lateral views, showing adequate patellar positioning. AP: anteroposterior

A postoperative radiograph showed the proper patellar positioning, as evidenced in Figure 5.

The postoperative rehabilitation protocol consisted of using an inguinal-malleolar brace for two weeks, with partial weight-bearing on the operated limb. After this period, passive range of motion was allowed up to 30 degrees, with gradual progression of weight-bearing. By the sixth week post-operation, the brace was completely removed, and weight-bearing was allowed based on tolerance. There were no observed neurovascular injuries or signs of infection (the intraoperative cultures were negative), and we also did not observe deficits in plantar flexion or eversion of the foot.

**Figure 6:**
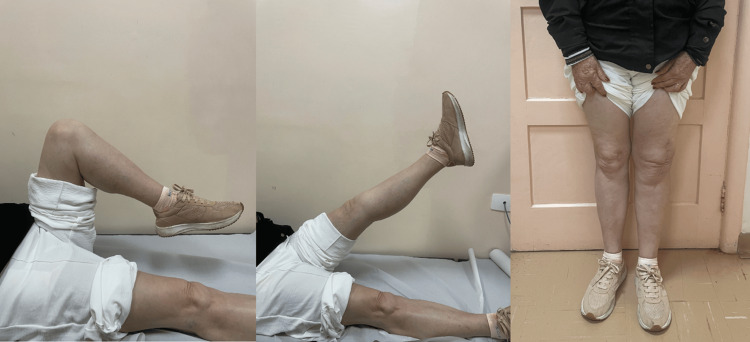
Range of motion and postoperative appearance at one year of follow-up.

In the outpatient follow-up, we used the Oxford Knee Score [12], with scores decreasing from 46 points at six weeks to 21 points at one year postoperatively. At the end of the first year, the patient demonstrated unassisted ambulation without limp, with full restoration of extensor mechanism competence, achieving a range of motion between 0 and 120 degrees, findings depicted in Figure 6.

## Discussion

Several techniques have been described for patellar tendon reconstruction following TKA; however, the results obtained are not yet completely satisfactory [13]. Although primary repair using isolated sutures or metallic anchors has shown favorable outcomes in extensor mechanism injuries in knees without arthroplasty [14], the same is not observed in those who have already undergone the procedure [3].

Most of the literature, as well as the most used technique currently, refers to the use of allografts, which have shown moderately successful outcomes [2,3,6,9]. However, a series of studies have also shown a significant number of patients experiencing residual deficits of the extensor mechanism, limited range of motion, and the need for ambulatory aids [6,10,15].

The use of synthetic materials has significantly increased in recent years, particularly due to greater commercial availability. Among the advantages of these devices, it is described that they can absorb load in the postoperative period, gradually transferring it to the repaired tissue during healing. Additionally, it highlights the absence of donor site morbidity (compared to autografts) and disease transmission risks (compared to allografts) [16]. However, the use of synthetic materials still faces challenges related to costs and access to the device, especially in developing countries, as well as risks of infection and tissue reaction in patients who have undergone previous TKA [17].

The use of autologous grafts for extensor mechanism reconstruction has been reported using various techniques [3,5]. Several types of grafts can be used, such as the semitendinosus and gracilis tendons, quadriceps flaps, gastrocnemius flaps, as well as free fascia lata grafts [3,8]. The use of grafts from the hamstring muscles is noted by some authors as more robust [18]; however, they may not be available in patients who have undergone multiple knee surgeries, and their length, thickness, and integrity may not be reliable [19].

The use of the peroneus longus tendon graft has been described and utilized in ligament reconstructions, especially of the anterior cruciate ligament [20-22]. However, its use in the reconstruction of the extensor mechanism is in its early stages; among the factors that support its use is the possibility of preparing a robust graft of the desired length and thickness [19].

In this case report, we present the approach to reconstructing the extensor mechanism in a post-TKA patient using the peroneus longus tendon. We observed a satisfactory postoperative outcome, with the return of the extensor mechanism, a wide range of motion, and ambulation without assistance. Furthermore, the use of the autograft minimizes costs, tissue rejection, or cross-infections, and there were no complications with the patient in the postoperative follow-up.

## Conclusions

The use of the peroneus longus tendon graft may be considered a viable alternative in the reconstruction of the extensor mechanism after TKA. The use of this graft allows for resolution in a single surgical procedure, minimizes costs, and reduces the chances of tissue rejection and cross-infection. Furthermore, it enables early rehabilitation. Further studies and graft comparisons should be conducted for better evaluation and validation of this method.
